# No effects of the theta-frequency transcranial electrical stimulation for recall, attention control, and relation integration in working memory

**DOI:** 10.3389/fnhum.2024.1354671

**Published:** 2024-02-19

**Authors:** Michał Ociepka, Suvarna Rekha Chinta, Paweł Basoń, Adam Chuderski

**Affiliations:** Department of Cognitive Science, Institute of Philosophy, Jagiellonian University, Kraków, Poland

**Keywords:** working memory, stimulation, transcranial alternate current stimulation (tACS), theta, theta frequency band

## Abstract

**Introduction:**

Recent studies have suggested that transcranial alternating current stimulation (tACS), and especially the theta-frequency tACS, can improve human performance on working memory tasks. However, evidence to date is mixed. Moreover, the two WM tasks applied most frequently, namely the n-back and change-detection tasks, might not constitute canonical measures of WM capacity.

**Method:**

In a relatively large sample of young healthy participants (*N* = 62), we administered a more canonical WM task that required stimuli recall, as well as we applied two WM tasks tapping into other key WM functions: attention control (the antisaccade task) and relational integration (the graph mapping task). The participants performed these three tasks three times: during the left frontal 5.5-Hz and the left parietal 5.5-Hz tACS session as well as during the sham session, with a random order of sessions. Attentional vigilance and subjective experience were monitored.

**Results:**

For each task administered, we observed significant gains in accuracy neither for the frontal tACS session nor for the parietal tACS session, as compared to the sham session. By contrast, the scores on each task positively inter-correlated across the three sessions.

**Discussion:**

The results suggest that canonical measures of WM capacity are strongly stable in time and hardly affected by theta-frequency tACS. Either the tACS effects observed in the n-back and change detection tasks do not generalize onto other WM tasks, or the tACS method has limited effectiveness with regard to WM, and might require further methodological advancements.

## Introduction

A key cognitive function is working memory (WM) – the brain network responsible for the active maintenance, transformation, and recall of information for the task at hand (Cowan, 2022). WM capacity positively predicts performance on multiple cognitive tasks such as stimulus discrimination (Troche et al., 2014), learning (Kaufman et al., 2009), language comprehension (Daneman and Merikle, 1996), reasoning (Chuderski, 2013), and problem solving (Wittmann and Süß, 1999). The recent decade has erupted with studies using various non-invasive interventions in order to improve WM, including neurofeedback (for review see Jiang et al., 2022) and transcranial magnetic stimulation (TMS; for review see Sloan et al., 2021). Another non-invasive method, growing in popularity, is transcranial electrical stimulation (tES). Evidence (see Thut et al., 2011; Abd Hamid et al., 2015; Santarnecchi et al., 2015; Herrmann et al., 2016; Galli et al., 2019; Lee et al., 2021; Grover et al., 2023; Wischnewski et al., 2023) suggested that it might be possible to improve WM using transcranial direct current stimulation (tDCS) and alternating current stimulation (tACS). Importantly, injected currents are so weak (typically 1000 mA−2000 mA) that they are barely detected and quickly habituated by participants, so placebo-controlled experiments (including so-called sham condition pretending real stimulation) can be easily implemented to test the effectiveness of various stimulation protocols, with great potential for both basic cognitive neuroscience research and clinical interventions.

As WM mechanisms are frequently associated with patterns of neural oscillations (Axmacher et al., 2010; Palva et al., 2010; Lisman and Jensen, 2013; Roux and Uhlhaas, 2014; Sauseng et al., 2019), the tACS technique has been especially popular in the ongoing attempts to enhance WM. A number of tACS studies (e.g., Hoy et al., 2015; Alekseichuk et al., 2016; Feurra et al., 2016; Santarnecchi et al., 2016; Tseng et al., 2016; Möller et al., 2017; Borghini et al., 2018; Pahor and Jaušovec, 2018; Misselhorn et al., 2020; Thompson et al., 2021; Kim et al., 2022; Palm et al., 2022; Park et al., 2022; Zeng et al., 2022; Grover et al., 2023; Kvašnák et al., 2023) targeted the fast rhythms, such as the gamma (>30 Hz) and, more rarely, the beta band (14 Hz−30 Hz), following the fact that coordinated fast oscillations were linked with active maintenance of particular objects in WM (e.g., Lisman and Jensen, 2013; Leszczyński et al., 2015). Several such studies reported positive effects of stimulation on WM (e.g., Hoy et al., 2015; Alekseichuk et al., 2016). The alpha band activity (8–13 Hz) was associated with distractor suppression in WM in MEG (Bonnefond and Jensen, 2012), EEG (Zhou et al., 2023) and TMS studies (Hamidi et al., 2009; Sauseng et al., 2009), but it was also related with coordination and controlled access (Klimesch, 2012), especially in visuo-spatial WM (Roux and Uhlhaas, 2014). The alpha band was targeted by tACS less frequently, and such studies reported its negligible effects on WM performance (e.g., Feurra et al., 2016; Borghini et al., 2018; Soutschek et al., 2022; Chen et al., 2023).

The largest number of tACS studies (e.g., Jaušovec and Jaušovec, 2014; Jaušovec et al., 2014; Meiron and Lavidor, 2014; Vosskuhl et al., 2015; Alekseichuk et al., 2016, 2017; Feurra et al., 2016; Santarnecchi et al., 2016; Kleinert et al., 2017; Borghini et al., 2018; Pahor and Jaušovec, 2018; Röhner et al., 2018; Tseng et al., 2018; Wolinski et al., 2018; Bender et al., 2019; Jones et al., 2019; Reinhart and Nguyen, 2019; Abellaneda-Pérez et al., 2020; Guo et al., 2021; Hosseinian et al., 2021; Sahu and Tseng, 2021; Biel et al., 2022; Draaisma et al., 2022; Hu et al., 2022; Soutschek et al., 2022; Zeng et al., 2022; Zhang et al., 2022; Grover et al., 2023; Rauh et al., 2023; Yang et al., 2023) targeted the theta band (4 Hz−7 Hz), which was proposed as the brain rhythm most strongly involved in encoding and maintaining the item sequences in WM (e.g., Lisman and Idiart, 1995; Jacob et al., 2018; Sauseng et al., 2019).

In one of the first attempts to stimulate the theta band frequency, Jaušovec et al. (2014) induced the current for 15 min at the individually adapted theta frequency, and then applied to his participants the forward and backward recall tasks as well as the 3-back task. The two recall tasks required repeating the sequence of items in either the same or the reversed order, respectively. The 3-back task required memorizing the last three items of a continuous stream of items, and responding each time the current item matched the item shown three items back. Depending on the stimulation location (left frontal, left parietal, right parietal), an improvement in each task was observed, as compared to the sham condition. A corresponding improvement for the forward and backward recall tasks was replicated by Vosskuhl et al. (2015) as a result of stimulating at individual theta frequency minus 1 Hz (i.e., presumably slowing down a key theta rhythm). Zhang et al. (2022) observed no increase for the visuospatial forward recall task when slowing the theta down, but noted an increase when tuning stimulation precisely to individual theta frequency (at around 6 Hz). Null effects were reported also by Feurra et al. (2016) for 5-Hz tACS. We are aware of no more replications of the theta stimulation effects on the working memory recall tasks in healthy adults. For the n-back task, enhancement effects of the theta frequency stimulation were suggested by Alekseichuk et al. (2016), Abellaneda-Pérez et al. (2020), and Biel et al. (2022) for 6 Hz, and Zeng et al. (2022) for 8 Hz.

Jaušovec and Jaušovec (2014; see also Reinhart and Nguyen, 2019) replicated the theta stimulation effect targeting individual theta frequency also for another WM task – the change detection task, in which a pattern of several items was masked and then replaced either by the same pattern or the pattern with a single item changed, and the participants had to detect the change. Resembling the Vosskuhl et al. (2015) findings, but in contrast with Zhang et al. (2022), Wolinski et al. (2018) found that stimulating at 4 Hz increased change detection for stimuli contralateral to the stimulation, while the 7-Hz protocol deteriorated change detection. Bender et al. (2019) reported a similar effect for 4 Hz, and Sahu and Tseng (2021) found it for 5 Hz. Analogous effects were observed for 5-Hz repetitive TMS in the visual (Riddle et al., 2020) and auditory matching task (Albouy et al., 2017).

However, other studies yielded null or at best mixed results regarding the theta-frequency tACS for the n-back (Pahor and Jaušovec, 2018; Röhner et al., 2018; Jones et al., 2019; Soutschek et al., 2022; Zeng et al., 2022; Yang et al., 2023) and change-detection task (Santarnecchi et al., 2016; Alekseichuk et al., 2017; Kleinert et al., 2017; Pahor and Jaušovec, 2018; Tseng et al., 2018; Zhang et al., 2022).

The above summary suggests that the majority of WM measures in the theta tACS studies so far comprised the n-back and change-detection tasks. However, even though the n-back task is frequently used in cognitive neuroscience (probably due to its perceptual and response simplicity), it is not a canonical WM task, because beyond WM maintenance it also requires rapid decision making (which relies on individual decisional criteria) and memory updating (which involves specific cognitive functions such as memory removal; see Ecker et al., 2014), and thus this task not always fully overlaps with more typical WM recall tasks (Kane et al., 2007; Schmiedek et al., 2009). Relatedly, the change detection task is mainly interpreted as a measure of the visual short-term memory buffer capacity (Cowan, 2022), and it can be easily turned into an iconic memory measure (Sligte et al., 2008), so the construct it taps might not be equivalent to general WM capacity (see Jastrzebski et al., 2021). By contrast, more canonical WM tasks require encoding, storage, and serial or free recall of a number of distinctive memory items (see Unsworth and Engle, 2007). So, more stimulation studies using typical recall tasks are needed to generalize the theta-frequency tACS effects onto a broader WM construct.

Moreover, WM capacity has frequently been associated with another two key functions – attention control and relational integration – which have never been targeted by the theta-frequency tACS thus far. Attention control is the ability for goal-directed processing that blocks goal-unrelated distraction (Kane and Engle, 2002). Capacious WM may reflect strong attention on items to be memorized, while ignoring the irrelevant stimuli. In this perspective, WM is viewed as a part of executive functions architecture underlying flexible, controlled cognition (McCabe et al., 2010; Diamond, 2013). Previous work suggested a key role of the theta rhythm for neural control and communication (Colgin, 2013), and several recent studies linked frontal midline theta to control within and over WM (e.g., Cavanagh and Frank, 2014; Berger et al., 2019; Ratcliffe et al., 2022). Thus, it was crucial to examine if processing on a task that taps into control over distraction can be modulated by stimulation at the theta frequency.

Relational integration reflects the ability to construct meaningful structures (sequences, relations) from single items held in WM (Oberauer and Lewandowsky, 2008). Tasks capturing relation integration strongly predict reasoning and problem solving and explain more or less the same variance that is measured by more typical WM tasks (Jastrzebski et al., 2020). Processing relations in the brain was also frequently linked to the theta rhythms (e.g., Brzezicka et al., 2011; Knowlton et al., 2012; Zhang et al., 2015), so theta band seemed the most promising target for relational integration stimulation.

Our general objective was to validate theta-frequency tACS modulation of WM, which is the most popular line of tACS-WM research. More specifically, our objective was to extend the existing tACS literature with regard to the three above discussed WM functions: storage and recall, attention control, and relational integration. To this aim we applied the theta-frequency stimulation to a relatively large sample of participants (*N* = 62), as compared to the sample sizes of tACS studies in the WM domain examined so far (*N*s ≈ 30). Using a within-subjects design, we applied the frontal and the parietal stimulation, and compared their effects to the sham condition. We chose a fixed theta frequency of 5.5 Hz, because the mean frequency applied in 47 separate experiments reported thus far equaled 5.47 Hz, which is close to the medium point of a typical theta band range (i.e., 4 Hz−7 Hz).

## Method

### Participants

Healthy adult participants were recruited via internet advertisements from a general population in Krakow, Poland. The age range of 38 women and 24 men was 18–29 years (*M* = 22.5, *SD* = 2.6). The participants had normal or corrected-to-normal vision and no history of neurological problems. During recruitment to the study and before each its session, each participant underwent a safety survey to make sure that there were no health issues that would prevent them from participating. The participants were informed that their data would be anonymous and they could interrupt the data collection and exit the laboratory at any moment. They signed a written consent form to participate and were paid the equivalent of 30 euros in local currency. Each participant was tested in a soundproof, dimly lit cabin, under the supervision of an experimenter. The study was approved by the local ethics board, and fully conformed with the Declaration of Helsinki.

### WM tasks

One task per each of three investigated WM functions was applied. In order to avoid that any differences in the potential stimulation effects between the tasks could depend on their visual characteristics, we made these characteristics similar across the tasks, with each task including colored geometric shapes. The tasks are described in the order they were attempted by participants.

**Figure 1:**
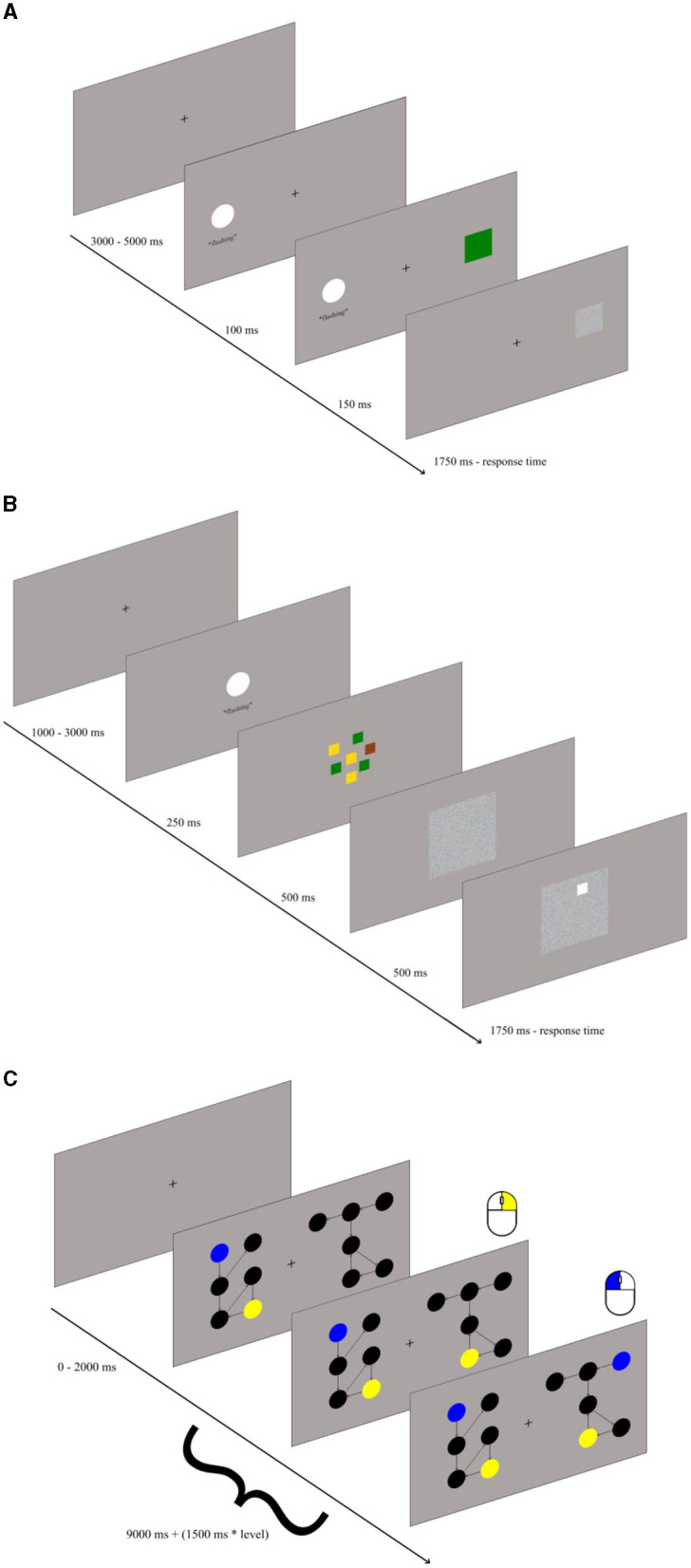
Illustration of the antisaccade **(A)**, recall **(B)**, and graph mapping task **(C)**.

*Attention control – the antisaccade task*: The antisaccade task is likely the most common and reliable test of attention control (Draheim et al., 2021). We adapted the task variant previously used in our laboratory (Jastrzebski et al., 2021), only substituting original arrows with colored squares. The task consisted of 100 trials. Each trial consisted of three events (see Figure 1A). First, a fixation point was presented at the center of the screen for between 3 s and 5 s (randomly, to avoid automatizing the task performance). Next, a rapidly flashing white circle (3 cm in size) was shown either on the left or right side of the screen for 0.25 s. Finally, 0.1 s after the circle onset, a 2-cm blue, green, or brown square was presented at the location opposite to the circle for 0.15 s and was then replaced by a mask. The visual angle from both the circle and the square to the fixation point was around 8 angular degrees. The task was to look away from the flashing square, to detect the color of the square, and to press a key associated with this color, as indicated by the color sticks attached. Participants were given 1.75 s to respond, otherwise the trial elapsed. The score in the task consisted of the proportion of correct responses out of 100.

*Storage and recall – the color recall task:* The recall task was designed specifically for the purposes of the current study. It also consisted of 100 trials. Each trial (see Figure 1B) started with the fixation point shown for 1 s to 3 s. Next, the rapidly flashing white circle was shown at the center of the screen for 0.25 s. With the circle offset, squares in yellow, green, and brown colors were shown centrally at random positions (in an area spanning 6 angular degrees). There were always from one to four squares per color, totaling to seven squares. After 0.5 s, the squares were replaced by a mask. Exactly 0.5 s after the mask onset, the white rim was shown at the location of one of the previously shown colored squares, and the task was to press, within 1.75 s, the key marked with the color stick matching its color. The task score was the proportion of correct responses out of 100.

*Relational integration – the graph mapping task*: We used the graph mapping task originally developed by Jastrzebski et al. (2023), which is a highly valid measure of relational integration. Each trial started with the fixation point shown for 0 to 2 s followed with main relational integration task. In this task two perceptually-different but relationally-isomorphic directed graphs were presented on the left- and right-hand side of the screen, with each graph consisting of black dots representing vertices and directed arrows representing edges (see Figure 1C). Each graph occupied area spanning around 6 angular degrees horizontally and vertically. Participants were required to match two vertices across the two graphs. Some vertices could be identified by the unique number of incoming and outgoing edges (its degree). Encoding the degree required representing a relation of the specific vertex with the number of its incoming and outgoing edges. Such a relation might be binary (e.g., “the vertex has one incoming edge”) or ternary (e.g., “one incoming and two outgoing edges”). Some other vertices had to be identified by representing the unique connections with the other vertices, such as “the vertex with one outgoing edge connects to the vertex with another two incoming vertices.” Therefore, the task required to integrate the relations between vertices and edges into a more complex graph structure. The two vertices to be mapped were highlighted in the left-hand graph with the blue and yellow color, respectively. The task was to click with the left mouse button on a vertex of the right-hand graph which mapped relationally onto the blue vertex in the left-hand graph, and to click with the right mouse button on a vertex of the right-hand graph which mapped onto the yellow vertex of the left-hand graph (the order of clicks was irrelevant). The participants were allowed (9 + 1.5^*^k)s for responding before the trial elapsed (where k = 1 to 5 represents level of difficulty of task). There were 36 trials. The task score was the proportion of correct responses, defined as the correct mapping of both the blue and the yellow vertex in a trial, out of 36 trials.

### Apparatus

**Figure 2:**
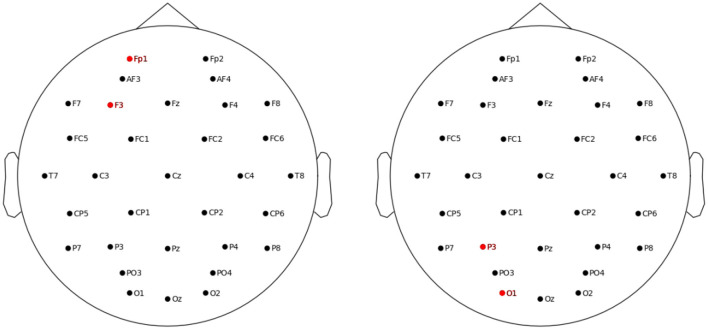
Location of the left frontal **(left panel)** and left parietal **(right panel)** stimulation electrodes.

The three tasks were controlled and displayed using a high-performance PC computer. The electrical stimulation was applied using the StarStim device (Neuroelectrics, Barcelona, Spain). To deliver the tACS, two 5 sq cm circular electrodes were used. Two stimulation layouts were applied (see Figure 2). For the frontal layout, the F3 was the stimulating electrode and the Fp1 was the return electrode (i.e., stimulation area including left dorsolateral prefrontal cortex, DLPFC). For the parietal layout, the respective electrodes were the P3 and the O1 (stimulation area including associative visual cortex and inferior parietal lobule, IPL). The F3 and/or P3 electrode were targeted by 18 studies (Jaušovec and Jaušovec, 2014; Jaušovec et al., 2014; Meiron and Lavidor, 2014; Feurra et al., 2016; Santarnecchi et al., 2016; Alekseichuk et al., 2017; Borghini et al., 2018; Pahor and Jaušovec, 2018; Röhner et al., 2018; Tseng et al., 2018; Jones et al., 2019; Reinhart and Nguyen, 2019; Abellaneda-Pérez et al., 2020; Hosseinian et al., 2021; Thompson et al., 2021; Biel et al., 2022; Hu et al., 2022; Rauh et al., 2023), which comprise the majority of theta-tACS studies on WM administered so far. Some of the above and 7 other studies targeted also the F4 and/or P4 electrodes, that is, right DLPFC and right IPL (Kleinert et al., 2017; Wolinski et al., 2018; Bender et al., 2019; Guo et al., 2021; Sahu and Tseng, 2021; Draaisma et al., 2022; Zhang et al., 2022). Because meta-analyses suggested that primarily the left DLPCF and IPL structures are associated with WM functions (Hill et al., 2014) and processing relations (Wertheim and Ragni, 2018), and the F3 and P3 electrodes comprised the most frequent choice among existing theta-tACS studies, we decided to target the F3 and P3 electrodes also in the current study.

### Procedure

Each participant visited the sound-proof, dimly-lit laboratory for three sessions, each lasting ~1 h. Two consecutive sessions were separated by at least 24 h, for any previous session effects to fade out. Each session started with mounting the electrode cap and the short training for the three tasks. Then, the three tasks were administered. Together with the antisaccade task onset, either a stimulation or the sham condition started. In the verum stimulation, the 2000-mA current was injected at 5.5 Hz frequency (i.e., the anode and cathode switched eleven times per second) for 20 min (i.e., below maximum 30 min stimulation time recommended for our tACS device), starting with the 30-s ramping up interval, and ending with 30-s fading out interval, with 19 min of full power stimulation in-between. The stimulation end co-occurred with the late stage of the recall task, so the antisaccade and the recall task were performed during stimulation, while the graph mapping task – just after the stimulation (as a recent meta-analysis suggests, offline tACS aftereffects are comparable to active stimulation; Grover et al., 2023). In the sham condition, there was only 30 s of ramping up after the antisaccade task onset, followed immediately by 30 s of fading out (no actual stimulation), in order to yield a similar initial subjective experience as in the verum stimulation. Half of participants received sham in the frontal layout, half – in the parietal layout. As a result of the procedure, three respective task scores were obtained in each of the three sessions, administered to each participant in the random order.

Before and after each session, the participants performed a vigilance check by observing a clock on the computer screen for 5 min. Five times per minute, the second hand “jumped” by 2 s instead of 1 second. The participants were required to focus attention on the second hand and press the key each time it jumped. The goal of introducing this task was to monitor the participants' level of attention during the session, as a drop in that level might affect potential stimulation gains. After each session, the participants (with one participant failing at this stage) reported qualitatively any of their negative subjective experience related to stimulation as well as they evaluated qualitatively its strength (from 0 = none via 5 = moderate up to 10 = very strong).

## Results

**Figure 3:**
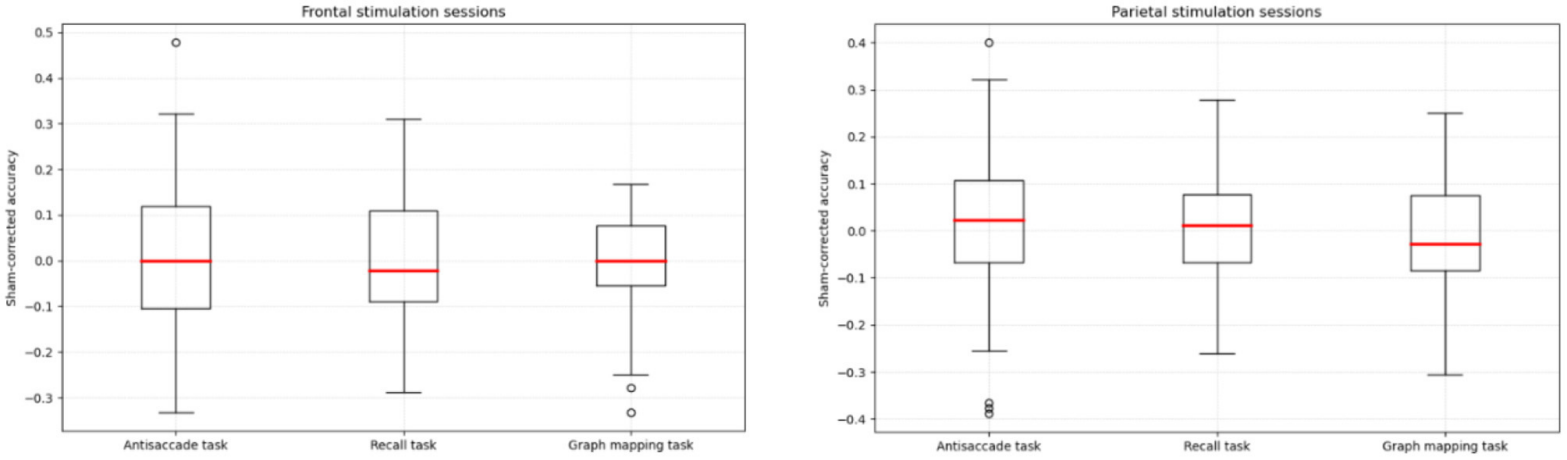
Sham-corrected accuracy (stimulation condition minus sham condition) in the antisaccade, recall, and graph mapping task, respectively, for the frontal and parietal verum stimulation sessions.

Table 1 presents the descriptive statistics for the three tasks and the three sessions. All the scores yielded normal distribution, with absolute values of both skew and kurtosis falling below 0.8. Figure 3 presents box-plots for sham-corrected performance in the frontal and parietal stimulation sessions.

**Table 1:** Mean scores (M), their standard deviations (SD) and ranges for the antisaccade, recall, and graph mapping task for the frontal and parietal verum stimulation sessions and the sham session.

**Task**	**Antisaccade**				**Recall**				**Graph mapping**			
**Session**	***M***	***SD***	**Min**.	**Max**.	***M***	***SD***	**Min**.	**Max**.	***M***	***SD***	**Min**.	**Max**.
Frontal	0.619	0.149	0.344	0.967	0.655	0.159	0.304	0.956	0.661	0.114	0.333	0.861
Parietal	0.626	0.154	0.222	0.889	0.649	0.159	0.304	0.978	0.673	0.092	0.472	0.889
Sham	0.617	0.153	0.211	0.867	0.647	0.148	0.348	0.938	0.676	0.109	0.417	0.889

To test whether the particular task scores differed significantly between the sessions, each score was submitted to repeated measures ANOVA with a single factor of session (frontal, parietal, sham). No effect of session was statistically significant: *F*_(2,122)_ = 0.097, *p* = 0.907, η^2^ = 0.002 for the antisaccade task, *F*_(2,122)_ = 0.145, *p* = 0.865, η^2^ = 0.002 for the recall task, and *F*_(2,122)_ = 0.672, *p* = 0.513, η^2^ = 0.010 for the graph mapping task. In order to confirm that these results can support the null hypothesis, we calculated the Bayes factor in favor of the null hypothesis, which yielded values of BF_01_ = 16.40, BF_01_ = 16.41, BF_01_ = 10.18, respectively, that is, each value provided strong support for the hypothesis that our tACS stimulation yielded no effect on any of task scores, as compared to the sham condition. Null stimulation effects were also found, each *p* > 0.50, after seven participants identified in Figure 2 as outliers (an absolute difference larger than *M* = 0.30 for a given task score observed between any stimulation session and the sham session) were excluded from the analysis.

**Figure 4:**
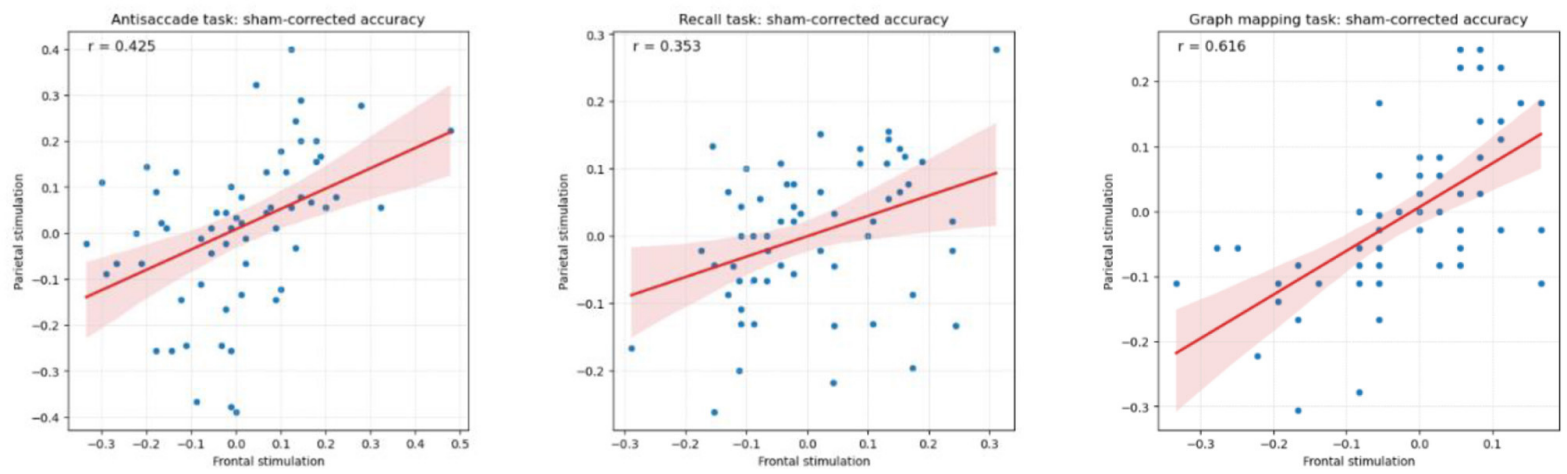
Pearson correlation between sham-corrected accuracy in the frontal and the parietal stimulation condition (respective stimulation condition minus sham condition) for the antisaccade, recall, and graph mapping task, showing substantial stability of performance in these two conditions.

A factor that needs to be accounted for in any test-retest design is the size of regression to the mean effect, that is, the tendency for people why scored externally low (high) in one session to score less low (less high) in another session (see Smoleń et al., 2018). In order to qualify the regression to the mean effect, we computed correlations between the given task's score in the sham session and the gain (a difference in that score between either the frontal or the parietal stimulation session and the sham session). The consecutive correlations equaled *r* = −0.555 (antisaccade, frontal), *r* = −0.546 (antisaccade, parietal), *r* = −0.332 (recall, frontal), *r* = −0.265 (recall, parietal), *r* = −0.473 (graph mapping, frontal), *r* = −0.693 (graph mapping, parietal), each *p* < 0.001, except for *p* = 0.037 for the parietal stimulation of recall. In consequence, we observed moderate to strong regression to the mean. Moreover, there was an analogous trend for the parietal stimulation gain to be large (small), if the frontal stimulation gain was large (small), and positive (negative) when the latter was positive (negative), with substantial correlations of *r* = 0.425 for the antisaccade, *r* = 0.353 for the recall, and *r* = 0.616 for the graph mapping task (see Figure 4).

There was a marginal drop in the clock task accuracy between pre-test (*M* = 0.991) and post-test (*M* = 0.989), *F*_(1,61)_ = 4.15, *p* = 0.046, η^2^ = 0.064, but accuracy was very high anyway and was not affected by the session factor, *F*_(2,122)_ = 1.50, *p* = 0.222. No effect pertained to time of reaction to the hand jumps, each *p* > 0.20. Individual accuracy never dropped below 0.90, and typically fell in the 0.95–1.0 range. Therefore, any potential gains from stimulation could not be counteracted by attention lapses.

Finally, out of 183 cases of reported subjective experience, in 47 (25.7%) cases the participants reported no negative experience, in 82 (44.8%) cases they reported weak experience (scores 1–3), in 37 (20.2%) cases – moderate experience (scores 4–6), and in the remaining 17 (9.3%) cases – strong experience (scores 7–10). Most of descriptions of experience referred to feelings of (in the order of appearance): tingling, smarting, heat, tickling, pain, lassitude, stinging, and current flow. In seven cases, some visual effects (flashes, dots, eye watering) were reported. There were significant and strong differences in subjective experience strength across the sessions, *F*_(2,120)_ = 17.99, *p* < 0.001, η^2^ = 0.230, with the frontal stimulation (mean score = 3.5) yielding a stronger experience than the sham (mean score = 2.4), *F*_(1,60)_ = 12.88, *p* < 0.001, and the sham yielding a stronger experience than the parietal stimulation (mean score = 1.7), *F*_(1,60)_ = 5.65, *p* = 0.021. The latter difference, however, was driven by the frontal sham (mean score = 2.9), while the parietal sham yielded comparable experience (mean score = 1.9) to the parietal stimulation, *F* < 1. The frontal sham and the frontal stimulation experience did not differ significantly, either, *F* < 1. The three estimates of experience strength (i.e., frontal, parietal, and sham) moderately correlated, with mean *r* = 0.432, each *p* < 0.001, but neither performance nor gains in any of the three tasks were significantly related with any of these subjective estimates or their differences. So, the subjective experience related with the procedure was primarily determined by the individual proneness to report such experience as well as by the area of the skull involved (stronger experience reported for the frontal than the parietal area). By contrast, the presence or absence of actual stimulation did not matter for the strength of subjective experience, and the latter was unrelated to the indices of performance on the WM tasks.

## Discussion

This study aimed to add to the unequivocal literature pertaining to effects of the theta-frequency alternating current stimulation on the WM performance, extending the range of examined tasks from primarily the n-back and change-detection tasks (neither being a canonical WM task) to three tasks that tap into central functions of working memory: attention control, serial recall, and relational integration. We controlled for the level of attentional vigilance during the sessions as well as obtained self-reports on subjective experience related to either verum or sham stimulation. Neither the frontal nor the parietal left hemisphere stimulation yielded any significant effect for the three WM tasks, despite the fact that for the present design our relatively large sample allowed for detecting effects larger than Cohen's f = 0.20 with 95% power, and effects larger than f = 0.15 with 80% power. Thus, we should have observed even medium effects if they existed. In consequence, this study provided evidence in line with studies that failed to observe clear effects of the theta frequency stimulation on working memory performance (e.g., Kleinert et al., 2017; Pahor and Jaušovec, 2018; Röhner et al., 2018; Jones et al., 2019; Soutschek et al., 2022). Our findings match also outcomes of a recent meta-analysis, suggesting no significant TMS effects on WM (Patel et al., 2020).

Another possibility may be that even though the n-back task and the change-detection task performance can indeed be sensitive to the theta-frequency stimulation (e.g., Alekseichuk et al., 2016; Tseng et al., 2018; Wolinski et al., 2018; Bender et al., 2019; Abellaneda-Pérez et al., 2020; Riddle et al., 2020; Sahu and Tseng, 2021; Zeng et al., 2022), this effect does not easily generalize onto the recall tasks (contrasting Jaušovec et al., 2014; Vosskuhl et al., 2015), as well as to attention control and relation integration tasks (no data available thus far).

One plausible explanation of the null results of our stimulation procedure is suggested by the strong effect of the regression to the mean. The fact that the performance in the sham session strongly but negatively predicted performance in the stimulation sessions, together with fact that after subtracting the sham performance the resulting difference in the frontal and parietal stimulation sessions correlated strongly positively, indicate that actually the individual performance across sessions was exceptionally stable. As the recall (Unsworth and Engle, 2007), the antisaccade (Draheim et al., 2021), and the graph mapping task (Jastrzebski et al., 2020) are known as highly reliable and valid markers of individual differences in WM capacity, scores on these tasks may reflect individual effectiveness of neurocognitive mechanisms underlying such capacity, which might be difficult to change with a relatively simple tACS intervention (see analogous failures to increase WM capacity and fluid intelligence using cognitive training protocols; Sala et al., 2019; Ripp et al., 2022). At the same time, the n-back and the change detection tasks, as less reliable and less indicative of individual differences in WM capacity (see Schmiedek et al., 2009; Jastrzebski et al., 2021), may be more sensitive to subtle improvements to the testing conditions resulting from tACS (e.g., improved attention focusing). These two tasks impose also simpler processing requirements (recognizing whether the current stimulus is the same as, or different from, 2–4 other simple stimuli), as compared to the recall tasks (reproduction of a list of stimuli in the correct order), the antisaccade task (going through a sequence of rapid events on the screen), and the graph mapping task (representation and transformation of a complex mental structure). With a lower complexity of a WM task, it might be more likely that the tACS interventions tap into the processes that translate into the final task score, while in more complex tasks only improving the coordination of an entire network of processes (and not a single mechanism) can affect the final score. Definitely, future studies of tACS effects on WM should take into account the requirement of various types of tasks applied to measure this construct.

This study had certain limitations. First, like most of other studies, we applied only a single stimulation session to our participants (i.e., did not successively repeat stimulation of the same skull area). Perhaps, like in some clinical areas of tACS intervention (e.g., Sprugnoli et al., 2021), multiple sessions are necessary to observe significant stimulation effects. However, at this stage of tACS research, combining large samples and sophisticated cognitive tasks with multiple sessions would be unfeasible practically. Second, perhaps cognitive performance of young healthy adults is close to ceiling, and thus difficult to further improve, so it might be easier to observe significant tACS effects in non-adult samples (children, older people) or in clinical populations. However, we were primarily interested in studying general effects of tACS. Third, we stimulated at the fixed frequency of 5.5 Hz, while using an individually adapted theta frequency might have yielded a different outcome. Future tACS studies of the recall, antisaccade, and relation integration tasks could address these limitations.

Concluding, this study examined a relatively large (as for a tACS study) sample of young healthy participants, drawn from a general population (not entirely students), tested with three different but canonical WM tasks, and used two different stimulation protocols (left frontal and left parietal), in a within-subjects design, controlling also for attentional vigilance and subjective experience. Nevertheless, we observed no reliable effect of any tACS protocol on WM performance. As the recent replication crisis in psychology and neuroscience have shown that null findings (given they were obtained with a sufficient power) may be comparably important to science as are significant effects, this study adds a substantial piece of data to our existing (still scarce) knowledge on the mechanisms of WM and ways to improve them. Even though a recent meta-analysis of transcranial electrical stimulation studies (see Grover et al., 2023) suggested a moderate optimism with regard to improving cognitive performance, much more data from various populations, tasks, stimulation protocols is needed to create a more complete picture of opportunities and limitations related with the tACS technology, and the current study may help in creating such a more complete picture.

## Data availability statement

The original contributions presented in the study are included in the article/Supplementary material, further inquiries can be directed to the corresponding authors.

## Ethics statement

The studies involving humans were approved by the Faculty Research Ethics Committee, Philosophical Faculty, Jagiellonian University Kraków. The studies were conducted in accordance with the local legislation and institutional requirements. The participants provided their written informed consent to participate in this study.

## Author contributions

MO: Writing – original draft, Conceptualization, Data curation, Investigation, Methodology, Software. SC: Investigation, Writing – review & editing. PB: Writing – review & editing, Visualization. AC: Writing – original draft, Conceptualization, Supervision, Formal analysis, Funding acquisition, Project administration, Validation.
